# Acute crystalline lens opacification following intracameral phenylephrine during retinal detachment surgery requiring unplanned phaco-vitrectomy

**DOI:** 10.1093/jscr/rjag978

**Published:** 2026-10-27

**Authors:** Ananth Ranjit, Panagiotis Tsoutsanis, Konstantinos Kaprinis

**Affiliations:** Department of Ophthalmology, Northern Care Alliance NHS Foundation Trust, Rochdale Infirmary, Whitehall St, Rochdale OL12 0NB, United Kingdom; Department of Ophthalmology, Northern Care Alliance NHS Foundation Trust, Rochdale Infirmary, Whitehall St, Rochdale OL12 0NB, United Kingdom; School of Medical Sciences, Faculty of Biology, Medicine and Healthy, The University of Manchester, Stopford Building, Oxford Road, Manchester M13 9PL, United Kingdom; Department of Ophthalmology, Northern Care Alliance NHS Foundation Trust, Rochdale Infirmary, Whitehall St, Rochdale OL12 0NB, United Kingdom

**Keywords:** ophthalmology, cataract surgery, vitreoretinal

## Abstract

Intracameral phenylephrine is commonly used during intraocular surgery to facilitate pupillary dilation. Previous reports have described transient anterior capsule and cortical lens discolouration following intracameral phenylephrine administration. We describe a case of acute crystalline lens opacification developing immediately following intracameral phenylephrine administration during pars plana vitrectomy for rhegmatogenous retinal detachment. The resultant loss of retinal visualization necessitated conversion to an unplanned phaco-vitrectomy. Vitreoretinal surgeons should be aware of this rare but potentially significant complication when using intracameral phenylephrine in phakic eyes.

## Introduction

Intracameral phenylephrine is frequently used during ophthalmic surgery to enhance pupillary dilation and improve intraoperative visualization. It is generally considered safe, although rare cases of anterior capsule and cortical lens discolouration have been reported [1]. We report a case of acute crystalline lens opacification following intracameral phenylephrine administration during retinal detachment surgery, resulting in loss of posterior segment visualization and necessitating unplanned lens extraction.

## Case report

A 72-year-old male presented with a superior rhegmatogenous retinal detachment in the right eye. Best-corrected visual acuity was 6/5 bilaterally. Slit-lamp examination demonstrated mild nuclear sclerosis that was not visually significant.

A 25-gauge pars plana vitrectomy was planned. Following insertion of the vitrectomy ports, intracameral phenylephrine 2.5% was administered because of inadequate pupillary dilation. Figure 1 demonstrates the status of the lens prior to administration of phenylephrine. Immediately following administration, dense anterior crystalline lens opacification developed, obscuring the retinal view (Fig. 2). Irrigation and anterior chamber washout failed to improve visualization. To ensure the rest of the procedure could go as planned, a decision was made to convert to an unplanned phacovitrectomy.

**Figure 1:**
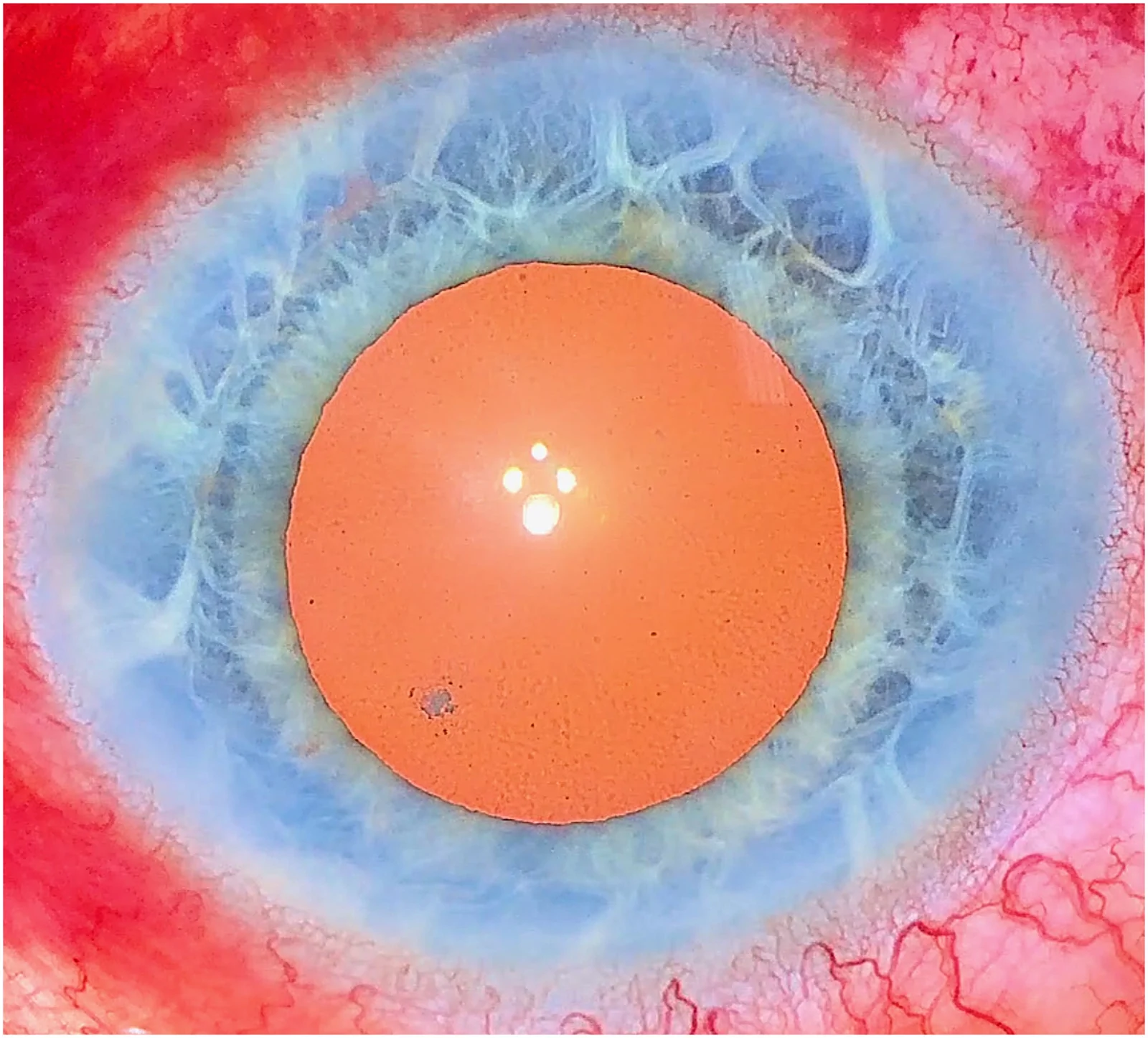
Clear crystalline lens immediately before intracameral phenylephrine administration.

**Figure 2:**
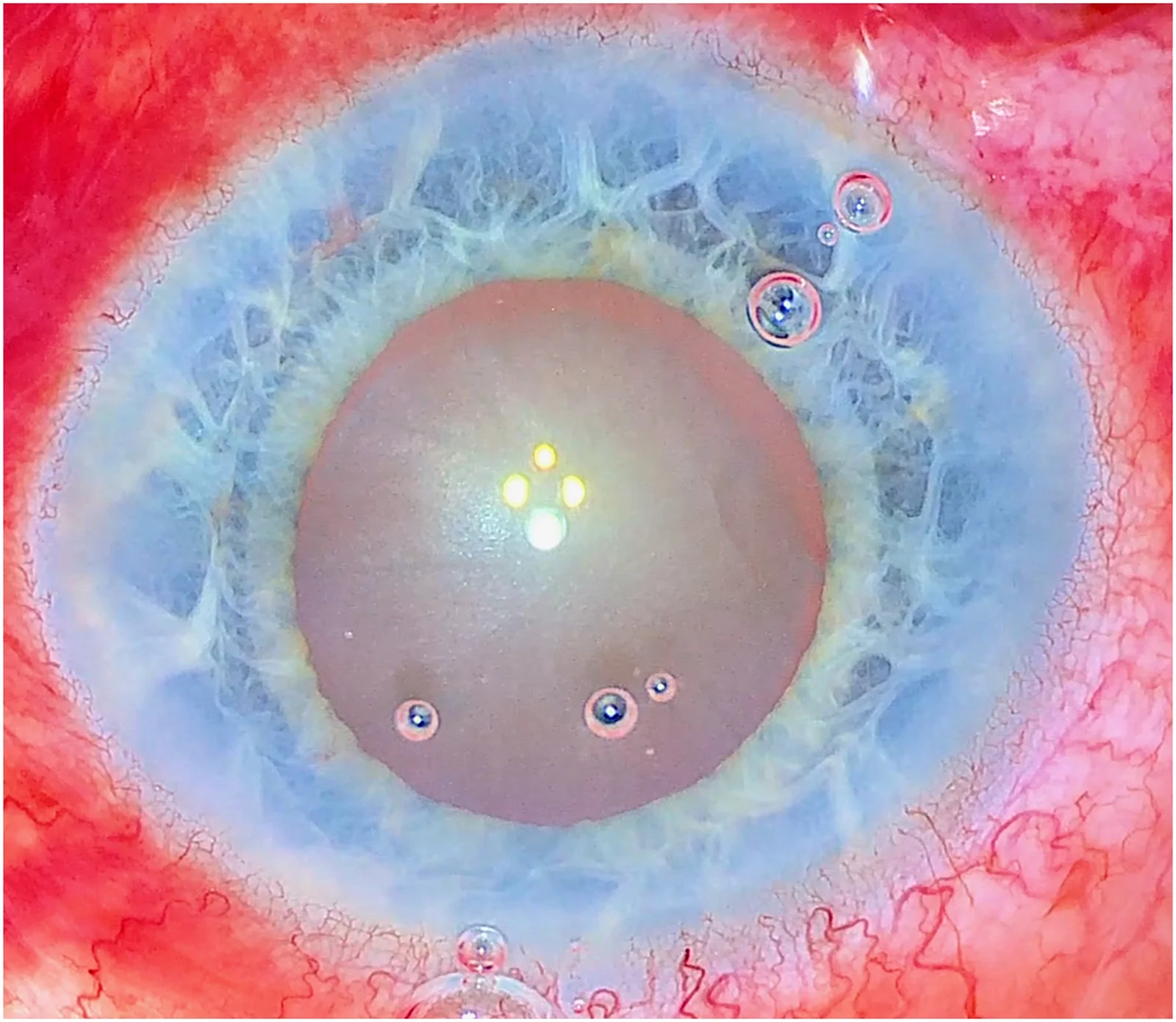
Acute development of anterior crystalline lens opacification after intracameral phenylephrine injection.

Phacoemulsification with intraocular lens implantation and Malyugin ring-assisted pupil expansion was performed, permitting completion of retinal detachment repair. Internal search identified a single retinal break adjacent to lattice degeneration. The patient had a vitrectomy, endolaser retinopexy, and C2F6 gas tamponade.

At six weeks postoperatively, unaided visual acuity in the right eye was 6/6, and the patient had a successful retinal detachment surgery.

## Discussion

Transient anterior capsule and cortical lens discolouration following intracameral phenylephrine has previously been reported. Tsatsos *et al.* reported characteristic spike-like crystalline lens staining developing shortly after intracameral phenylephrine injection, though it did not impact surgical management.

In contrast, our patient developed dense crystalline lens opacification that resulted in complete loss of retinal visualization and necessitated conversion to phacovitrectomy. To our knowledge, reports describing phenylephrine-associated lenticular changes severe enough to alter vitreoretinal surgical management remain limited.

An association between phenylephrine exposure and lenticular opacification has also been demonstrated experimentally. Claoué reported reversible lens opacification following topical 10% phenylephrine administration in mice, although the proposed mechanism in this model was transcorneal dehydration and its relevance to intracameral administration within clinical context remains uncertain [2]. Lockington *et al.* demonstrated that phenylephrine preparations contain higher concentrations of free radicals than other commonly used intracameral agents [3]. Oxidative stress may therefore represent a plausible mechanism for acute lenticular alteration. Tsatsos *et al.* additionally suggested that the duration of phenylephrine exposure may influence the degree of lens staining observed [1].

This case highlights an important consideration for vitreoretinal surgeons operating on phakic eyes. Although cataract progression is a recognized consequence of pars plana vitrectomy, acute lenticular opacification immediately following intracameral phenylephrine administration may result in loss of posterior segment visualization and necessitate unplanned removal of lens.

## Conclusion

Acute crystalline lens opacification is a rare complication of intracameral phenylephrine administration. In severe cases, loss of retinal visualization may necessitate conversion to phacovitrectomy to permit completion of retinal detachment repair. Surgeons should be aware of this complication when considering intracameral phenylephrine in phakic eyes.
